# *Leishmania* Parasite: the Impact of New Serum-Free Medium as an Alternative for Fetal Bovine Serum

**DOI:** 10.52547/ibj.25.5.349

**Published:** 2021-08-29

**Authors:** Sima Habibzadeh, Delaram Doroud, Tahereh Taheri, Negar Seyed, Sima Rafati

**Affiliations:** 1 Department of Immunotherapy and *Leishmania* Vaccine Research, Pasteur Institute of Iran, Tehran, Iran;; 2 Department of Biotechnology, Faculty of Advanced Science and Technology, Tehran Medical Science, Islamic Azad University, Tehran, Iran;; 3 Quality Control Department, Production and Research Complex, Pasteur institute of Iran, Tehran, Iran

**Keywords:** Growth rate, L. major, L. tarentolae, Serum-free medium, PpSP15-EGFP protein

## Abstract

**Background:**

Flagellated protozoan of the genus *Leishmania* is the causative agent of vector-borne parasitic diseases of leishmaniasis. Since the production of recombinant pharmaceutical proteins requires the cultivation of host cells in a serum-free medium, the elimination of FBS can improve the possibility of large-scale culture of *Leishmania *parasite. In the current study, we aimed at evaluating a new serum-free medium in *Leishmania *parasite culture for future live *Leishmania* vaccine purposes.

**Methods:**

Recombinant *L. tarentolae* secreting PpSP15-EGFP and wild type *L. major* were cultured in serum-free (CSFM) and serum-supplemented medium. The growth rate, protein expression, and infectivity of cultured parasites in both conditions was then evaluated and compared.

**Results:**

Diff-Quick staining and epi-fluorescence microscopy examination displayed the typical morphology of* L. major *and* L. tarentolae-*PpSP15-EGFP promastigote grown in CSFM medium. The amount of EGFP expression was similar in CSMF medium compared to M199 supplemented with 5% FBS in flow cytometry analysis of *L. tarentolae*-PpSP15-EGFP parasite. Also, a similar profile of PpSP15-EGFP proteins was recognized in Western blot analysis of *L. tarentolae-*PpSP15-EGFP cultured in CSMF and the serum-supplemented medium. Footpad swelling and parasite load measurements showed the ability of CSFM medium to support the *L. major* infectivity in BALB/C mice.

**Conclusion:**

This study demonstrated that CSFM can be a promising substitute for FBS supplemented medium in parasite culture for live vaccination purposes.

## INTRODUCTION

Leishmaniasis is a global complex vector-borne protozoan parasitic disease caused by an obligate intracellular parasite of the genus *Leishmania. *The parasite is transmitted in the metacyclic form to mammalian hosts by the bites of the infected female phlebotomine sand flies during their blood meal^[^^1^^]^. This neglected disease is widespread in 98 countries in some parts of the tropical and subtropical areas with a world annual incidence of about 0.7–1 million individuals and 26,000 to 65,000 deaths and nearly 350 million people at risk of this infectious disease^[^^2^^]^. There are different clinical manifestations of the disease based on the complex interplay between genetic and host immune response and also parasite and vector species^[^^3^^-^^5^^]^, which mainly appears as cutaneous leishmaniasis (the most common form), visceral leishmaniasis (known as kala-azar), and mucocutaneous leishmaniasis caused by different* Leishmania* species^[^^6^^,^^7^^]^. Leishmania parasite has a digenetic life cycle in the susceptible vertebrate host and insect vector as non-flagellated amastigotes and motile flagellated extracellular promastigotes, respectively^[^^8^^,^^9^^]^. Amongst different *Leishmania* species, *L. tarentolae *is a parasite of lizards and nonpathogenic to humans. This parasite represented as an important tool for recombinant protein production due to the numbers of its characteristics, including mammalian-like N-glycosylation patterns in addition to high similarity of post-translational modifications^[^^10^^]^. Furthermore, there are other advantages offered by this parasite, comprising a short 6-10-hour generation time in the culture medium, a simple and an affordable nutrient requirement that is less expensive than other mammalian cells, a high growth rate, and easy to work for the production of recombinant proteins with capability to rich an industrial production scale in bioreactors^[^^11^^,^^12^^]^. *L. tarentolae* has the potential and already demonstrated as an efficient approach for developing a secure and an effective live prophylactic vaccine against *Leishmania* infections^[^^13^^,^^14^^]^. It has been reported that recombinant *L. tarentolae* secreting PpSP15 (a 15-kDa immunogenic salivary protein from the *Phlebotomus papatasi* sand fly) can act as a new promising vaccine strategy, which induces protective immunity against *L. major* infection in BALB/c mice^[^^15^^,^^16^^]^.

Over the past decades, different kinds of media have been used for the cultivation of *Leishmania* parasite, which is mainly categorized into two principal groups: semi-solid biphasic media and liquid monophasic media, many of which, such as RPMI-1640, M199, BHI, and NNN media and Schneider’s Drosophila need enrichment by adding the appropriate amounts of blood or FBS as a common essential supplement to stimulate cell division and support the long-term growth of *Leishmania* promastigotes^[^^17^^,^^18^^]^. FBS has important components such as a large number of growth factors containing hormones, vitamins, transporter proteins, and adhesion factors, which all are crucial for the continuous maintenance and propagation of the cultured parasite^[^^19^^,^^20^^]^.

Despite the positive effects of FBS in cell culture, the presence of FBS represents numerous ill-defined properties that introduce significant limitations, such as the quantitative and qualitative differences in compounds in each batch and lack of reliable sources for developing countries involved with leishmaniasis as well as a growing concern about sacrificing the animal during the serum collection^[^^21^^,^^22^^]^. More importantly, serum-supplemented media, due to the possibility of microbial contaminants, can lead to problematic issues with pharmaceutical products for human therapy and vaccine development process^[^^23^^]^. Therefore, a reliable substitute for animal serum is required for the cultivation of host cells in diverse metabolic and biochemical studies and is a considerable necessity for laboratory research, vaccine, and drug production^[^^24^^]^.

The importance of cell culture in serum-free media is widely recognized today. The serum-free cultivation of *Leishmania* parasites is cost-effective and improves the large-scale production for these parasites^[^^25^^]^. So far, several attempts have been made to find appropriate substitutes for FBS by developing a variety of substances such as animal-derived substances^[^^26^^,^^27^^]^, soy protein isolate^[^^28^^]^, and the mixture of peptone and diverse extracts^[^^19^^,^^30^^]^, as well as some defined serum-free media^[^^30^^,^^31^^]^.

 In the present work, we developed a simple, easy handling, monophasic serum-free medium, named CSFM, to demonstrate its potential for the cultivation of live non-pathogenic recombinant* L. tarentolae* secreting Ppsp15-EGFP as a vaccine target and pathogenic form of *Leishmania* (*L. major*). Also, the expression of PpSP15-EGFP secretory protein and the infectivity of *L. major* parasite in a susceptible BALB/c mice model were confirmed in the presence of this new serum-free medium. 

## MATERIALS AND METHODS

**Animals**

Female BALB/c mice with the age of 6-8 weeks old and body weight of almost 20 g were purchased from breeding stock maintained at the Pasteur Institute of Iran, Tehran, Iran. Animals were kept in plastic cages under conditioned animal care facility with 12 hours light-dark cycles with free access to food and water.

**Media, reagents, and chemicals**

Double distilled water was applied to prepare all the solutions. The cell culture reagents, including M199 medium, Minimum Essential Medium Eagle, MEM non-essential amino Acid solution (100×), MEM amino acids (50×), BME vitamins solution (100×), L. glutamine, gentamicin, hemin, HEPES, D-glucose, and sodium bicarbonate were purchased from Sigma-Aldrich (Sigma, Deisenhofen, Germany) and FBS from Gibco^®^ (Life Technologies, Germany). SYBR green for real-time PCR was obtained from Qiagen (Germany), and GF-1 DNA extraction kit was provided by Vivantis Technologies (Malaysia). The materials needed for Western blot analyses, including BSA, diaminobenzidine, Tween 20, and SDS, were provided by Sigma-Aldrich. Acrylamide and anti-GFP-HRP goat polyclonal antibody were procured from Merck and Acris Antibodies GmbH (Herford, Germany), respectively. 

**Media preparation**

***Medium CSFM***

The formulation of the CSFM medium developed for the cultivation of pathogenic and non-pathogenic form of leishmania is shown in Table 1. The medium was prepared by mixing powders in 90 ml of distilled water; the pH was adjusted to 7.2 with NaOH of 10 M. After adding other supplements and adjusting the volume to 100 ml with distilled water, the medium was sterilized via a 0.22-µm nitrocellulose membrane (Millipore, Merck, Germany) and stored in the dark at 4 °C, due to the presence of hemin. This serum-free medium contained salt, sugar, hemin, essential and non-essential amino acids, and a high concentration of vitamins that are critical for the parasite growth. 

***Medium 199***

According to Table 1, M199 was provided as a standard comparative medium by dissolving 1.47 g of M199 powder and 0.22 g of sodium bicarbonate in 90 ml of distilled water. After pH adjustment to 7.2, the mixture was enriched with 5% heat-inactivated FBS, and the other additives were added. The medium volume was brought up to 100 ml and then sterilized by passing through 0.22-μm filters. 

**Parasite culture**

Three types of *L. tarentolae*, including the wild-type *L. tarentolae* Tar II (ATCC 30.267) strain, EGFP-expressing *L. tarentolae* (*L. tarentolae*-EGFP)^[^^32^^]^, and recombinant *L. tarentolae*-PpSP15-EGFP^[^^15^^]^ (as previously available), as well as wild-type *L. major *strain (MRHO/IR/75/ER) were used. At the first step, the promastigotes were cultured at 26°C in M199 supplemented with 5% heat-inactivated FBS.

**Growth determination**

The recombinant *L. tarentolae*-PpSP15-EGFP, which was initially cultured in M199 with 5% FBS, were collected by centrifugation at 5.9 g at 26 °C for 10 min and washed once with 10 ml of sterile PBS (137 mM of NaCl, 0.25 mM of KCl, 1.75 mM of KH_2_PO_4_, and 8 mM of Na_2_HPO_4_, pH 7.2) to remove FBS. The logarithmic phase was adjusted to a final concentration of 1 × 10^7^ /ml^-1 ^parasite and cultured in two 25-cm^2^ cell culture flasks filled with 10 ml of CSFM and another flask containing 10 ml of M199 5% FBS as control and incubated at 26 °C. Parasite growth was monitored qualitatively and quantitatively by daily microscopic observations for the appearance and mobility of the parasites and counting the promastigotes. The number of parasite was determined by a hemocytometer slide, and the optical density evaluation was performed by a spectrophotometer at 600 nm. The whole experiments were repeated three times in parallel.

**Fluorescence microscopy and flow cytometry analysis**

GFP expression was evaluated in *L. tarentolae*-PpSP15-EGFP promastigotes cultured in CSFM and M199 5% FBS by epifluorescence microscopy (Nikon, E 200, ACT-1 software, Digital sight Camera, Japan) and flow cytometry. In parallel, *L. tarentolae*-EGFP and *L. tarentolae* wild type were included as positive and negative controls, respectively. After washing the parasites with PBS, the presence of green fluorescent protein (EGFP) was determined under a fluorescence microscope. The percentage of EGFP protein expression in the *L. tarentolae*-PpSP15-EGFP was examined using flow cytometry (FACS caliber flow cytometer), recording 50,000 events from each sample.

PpSP15-EGFP protein expression of *L. tarentolae*-PpSP15-EGFP cultured in both CSFM and M199 FBS 5% media. The stationary and logarithmic phase supernatant of *L. tarentolae*-PpSP15-EGFP and also the pellet of *L. tarentolae*-EFGP (positive control) and *L. tarentolae* wild type promastigotes (negative

**Table 1:** Composition of the CSFM and the Medium 199

**CSFM**		**Medium 199 with 5% FBS**	
**Components/100 ml**	**Amount**	**Components/100 ml**	**Amount**
Minimum Essential Medium Eagle	1.04 g	Medium 199	1.47 g
Sodium Bicarbonate	0.22 g	Sodium bicarbonate	0.22 g
MEM non-essential amino acid solution (100x)	1 ml	Heat-inactivated FBS	5 ml
HEPES	40 mM	Adenosine (10 Mm)	0.1 mM
MEM amino acids (50× )	2 ml	HEPES	40 mM
L. glutamine	1 mM	L. glutamine	1 mM
Hemin	0.5 µg/ml	Gentamicin	100 µg/ml
BME vitamins 100× solution	1 ml	Hemin	0.5 µg/ml
D-glucose	0.45 g		

**Western blotting**

Western blot analysis was applied to assess the control) were obtained after centrifuging at 0.8 g for 10 min. The supernatant of *L. tarentolae*-PpSP15-EGFP was concentrated (∼1:5) under vacuum by a Freeze Dryer device (Christ L-1, Alpha 2–4, Germany), electrophoresed by 12.5% SDS-PAGE polyacrylamide gel (Bio-Rad). For electro blotting, the protein contents were transferred onto a nitrocellulose membrane (Schleicher and Schuell Bioscience, Dassel, Germany). After overnight blocking with a blocking buffer (TBS with 0.1% Tween 20 and 2.5% BSA) at 4°C and three washes, the anti-EGFP antibody (GFP-HRP polyclonal antibody, 1:5000 v/v Acris antibodies GmbH) was used for two hours at room temperature. Finally, 3,3^ˈ^-diaminobenzidine (Sigma-Aldrich) was applied as a substrate to detect the EGFP and PpSP15 protein bands.

***L. major*** **parasite growth and animal infection**

Evaluation of* L. major *parasite growth in CSFM medium and M199 5% FBS was determined similarly as described above. Afterward, the infectivity assessment was performed by the subcutaneous injection of 2 × 10^6^/50 µl stationary phases of *L. major* parasites into the left footpad of susceptible female BALB/c mice (three animals per group). The mice were categorized into the following groups: group 1, infected by stationary phase *L. major* promastigotes cultured in M199 5% FBS and Group 2, the mice infected by *L. major* promastigotes grown in the CSFM medium. Footpad swelling was measured starting one week after infection with a metric caliper by subtraction of total thickness and wideness in infected footpad (left) from uninfected (right) footpad and followed up to seventh weeks post infection. Parasite burden quantification was carried out by sacrificing the infected BALB/C mice at 7^th ^week post infection by *L. major* parasite cultured in the CSFM medium and M199 5% FBS. Lymph node from each infected footpad was homogenized in sterile PBS 1×, and extraction of genomic DNA was performed by GF-1Tissue DNA Extraction Kit. After the determination of DNA concentration using a Nanodrop (ND-1000, USA), the absolute copy number of the kinetoplastid minicircle region of *L. major* parasite was evaluated in 15 ng of DNA by real-time PCR (Applied Biosystem 7500, USA) using specific forward and revers primers, named RV1 (5̍-CTTTTCTGGTCCCGCGGGTAGG-3̍) and RV2 (5̍-CCACCTGGCCTATTTTACACCA-3̍) for quantification of parasite load. The standard curve was drawn using six times dilution of the standard genomic DNA of* L. major* parasite (4m × 10^7^ cell ml^–1^). Each PCR reaction included 2.5 μl of genomic DNA sample (6 ng/μl), 5 pmol of each RV1 and RV2 primer, and 10 μl of SYBR Green PCR master mix (Qiagen) in a 20 μl total volume. The real-time PCR conditions were accomplished with following order: 95 °C for 5 min, 40 cycles of 95 °C for 15 s, 30 s at 58 °C, and 72 °C for 40 s in duplicate as previously reported^[^^13^^]^. 

**Statistical analysis**

For statistical analysis, (GraphPad Prism7 Software (USA.) and *t*-test were applied. Flow cytometry results were evaluated using FlowJo software. A *p* value less than 0.05 was considered statistically significant for all the experiments, and data were shown as mean ± standard deviation.

**Ethical statement**

The above-mentioned sampling protocols were approved by the Institutional Animal Care and Research Advisory Committee of the Pasteur Institute of Iran according to ethical guidelines (ethical code: 958432). 

## RESULTS

**Growth assessment of** ***L. tarentolae*** **-PpSP15-EGFP**

**Fig. 1:**
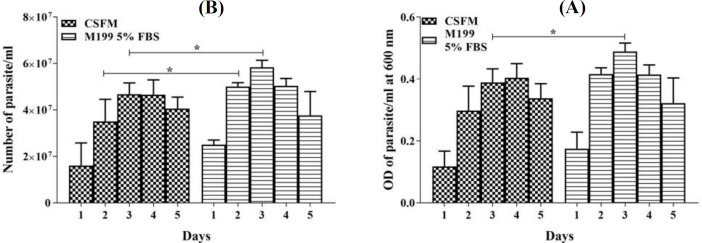
Growth curves of the *L. tarentolae*-PpSP15-EGFP parasite cultured in two different media with 1× 10^7^/ml inoculation in 25-cm^2^ flasks at 26 °C for a five-day period. (A) Growth rate evaluation of *L. tarentolae*-PpSP15-EGFP parasites (parasite count/ml) in CSFM medium compared to M199 5% FBS. (B) Growth rate evaluation of *L. tarentolae*-PpSP15-EGFP parasites (optical density at 600 nm/ml) in CSFM and M199 5% FBS. The experiment was repeated three times, and values are pooled in the Figure. Bars show the mean ± SD. The significant difference in parasite growth rate for different media was determined by *t*-test analysis and demonstrated as asterisk at the indicated time points (*p* < 0.05 denoted as ^*^). At the time points that are not marked with the asterisk, no significant difference was observed (*p* > 0.05)

The growth behavior of the recombinant *L. tarentolae*-PpSP15-EGFP parasite examined microscopically in CSFM medium was compared to the conventional M199 with 5% FBS. As it is shown in Figure 1A, the CSFM medium like M199 with 5% FBS can support the growth of *L. tarentolae*-PpSP15-EGFP parasite, just a slightly significant difference was observed at 2^nd ^and 3^rd^ days after inoculation (*p* < 0.05); however, the level of growth in other days was similar, and no significant differences were detectable (*p* > 0.05). In addition, the optical cell density similar to microscopic analysis showed a significant difference at 3^rd^ day (*p* < 0.05, Fig. 1B). Daily microscopic observation of parasite motility during culture was performed, and the parasites were found in normal size with appropriate mobility. These results described the ability of the CSFM medium to support the propagation of *L. tarentolae*-PpSP15-EGFP parasite similar to M199 with 5% FBS. 

**Evaluation of GFP expression**

**Fig 2:**
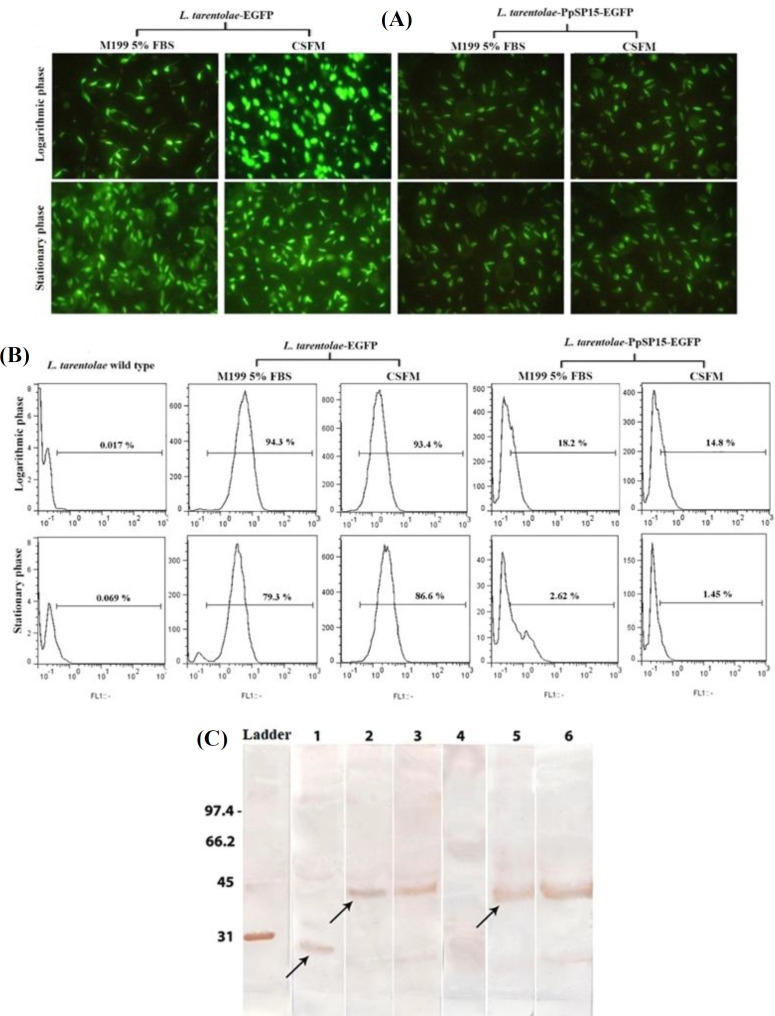
Determination of EGFP expression in CSMF medium in comparison with M199 supplemented with 5% FBS. (A) Assessment of EGFP expression by epifluorescence microscopy, indicating the EGFP expression of *L. tarentolae*-EGFP and *L. tarentolae*-PpSP15-EGFP promastigotes in the logarithmic and stationary phases in both CSFM and M199 5% FBS media. (b) Flow cytometry analysis of recombinant *L. tarentolae*-PpSP15-EGFP (right panel), *L. tarentolae* EGFP as a positive control (middle panel), and the *L. tarentolae *wild type as a negative control (left panel) using a FITC detector in CSFM and M199 5% FBS at logarithmic (upper panel) and stationary (lower panel) phases. (C) Western blot analysis to confirm the expression of PpSP15-EGFP by recombinant *L. tarentolae*-PpSP15-EGFP parasite in CSFM and M199 with 5% FBS using an anti-GFP antibody. A 42-kDa band relating to the expression of PpSP15-EGFP protein in concentrated supernatant of *L. tarentolae*-PpSP15-EGFP parasite was detected in M199 with 5% FBS (lanes 2 and 3) and CSFM medium (lanes 5 and 6), respectively at logarithmic and stationary phases. A 27-kDa band indicating EGFP protein in the* L. tarentolae*-EGFP parasite (lane 1) and wild type form of *L. tarentolae* parasite as negative control was cultivated in M199 with 5% FBS (lane 4)

Qualitative and quantitative GFP expression pattern were used to analyze and compare the EGFP expression of *L. tarentolae*-PpSP15-EGFP and *L. tarentolae*-EGFP cultured in CSFM and M199 with 5% FBS by epifluorescence microscopy and flow cytometry, respectively. As shown in Figure 2A, both media represented similar fluorescence related to the EGFP expression of *L. tarentolae*-PpSP15-EGFP and *L. tarentolae*-EGFP using microscopic observation at different growth phases (logarithmic and stationary). Also, flow cytometry analysis demonstrated the same level of EGFP expression by parasites cultured in CSFM compared to M199 with 5% FBS. The fluorescent intensity of the *L. tarentolae*-PpSP15-EGFP parasite was lower than that of the *L. tarentolae*-EGFP parasite, which may be due to the presence of PpSP15 before* egfp* gene (Fig. 2B).

**Western blotting and PpSP15-EGFP expression**

Production of PpSP15- EGFP was analyzed using Western blot at the logarithmic and stationary growth stages in the concentrated supernatant of *L. tarentolae*-PpSP15-EGFP parasite cultured in CSFM and M199 with 5% FBS. A band with a molecular weight of 42 kDa, due to PpSP15-EGFP protein production, was observed in *L. tarentolae*-PpSP15-EGFP parasite grown in CSFM and M199 with 5% FBS at both phases (Fig. 2C, lanes 2, 3, 5, and 6). These results confirmed the ability of the CSFM medium to support the *L. tarentolae*-PpSP15-EGFP parasite to express the PpSP15-EGFP protein. The* L. tarentolae*-EGFP as positive control expressed EGFP with a molecular weight of 27 kDa, and *L. tarentolae* wild-type parasite as negative control are shown in Fig. 2C (lanes 1 and 4, respectively).As shown in Fig 2A, the fluorescence microscopy confirmed the EGFP expression in *L.* *tarentolae*-EGFP and *L. tarentolae*-PpSP15-EGFP promastigotes in the logarithmic and stationary phases using both CSFM and M199 5% FBS media. Also, the flow cytometry analysis of recombinant *L. tarentolae*-PpSP15-EGFP (Fig 2B, right panel), *L. tarentolae* EGFP strains as positive controls (Fig. 2B, middle panel) and the *L. tarentolae* wild type as a negative control (left panel) using FITC detector in CSFM and M199 5% FBS at logarithmic (upper panel) and stationary (lower panel) phases indicated the successful expression of EGFP in positive strains. Western blot analysis also confirmed the expression of PpSP15-EGFP by recombinant *L. tarentolae*-PpSP15-EGFP parasite in CSFM and M199 with 5% FBS using an anti-GFP antibody. A 42-kDa band relating to the expression of PpSP15-EGFP protein in concentrated supernatant of *L. tarentolae*-PpSP15-EGFP parasite was detected in M199 with 5% FBS (lanes 2 and 3) and CSFM medium (Fig. 2C, lanes 5 and 6), respectively at logarithmic and stationary phases. A 27-kDa band indicating EGFP protein in the *L. tarentolae*-EGFP parasite (Fig. 2C, lane 1) and wild-type form of *L. tarentolae* parasite as negative control was cultivated in M199 with 5% FBS (Fig. 2C, lane 4).

**Monitoring** **of** ***L. major*** **growth** **and** **infectivity**

**Fig. 3:**
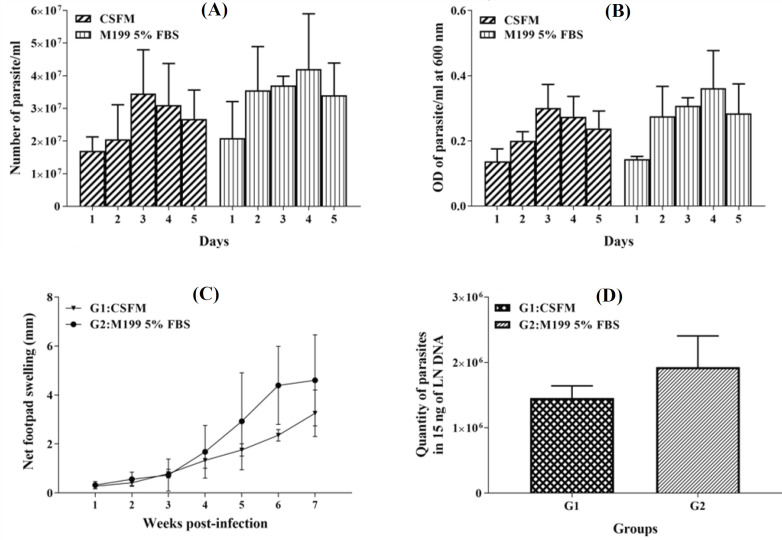
Examination of growth behavior of *L. major *parasite, footpad swelling measurement, and parasite load quantification by real-time PCR. (A) *In vitro* comparative growth analysis of *L. major* in CSFM and M199 with 5% FBS by cell counting. Error bars show the mean ± SD. (B) Growth curve achieved in the CSFM and M199 with 5% FBS using optical density determination at 600 nm. (C) Comparison of footpad swelling in mice infected with *L. major* promastigotes cultured in CSFM and M199 with 5% FBS up to 7^th^ week. (D) Parasite quantification in the lymph nodes of BALB/c mice following infection with *L. major* parasites cultured in CSFM and M199 with 5% FBS. The error bars demonstrate the standard deviation of the mean and the data analyzed by the *t*-test

**Fig. 4:**
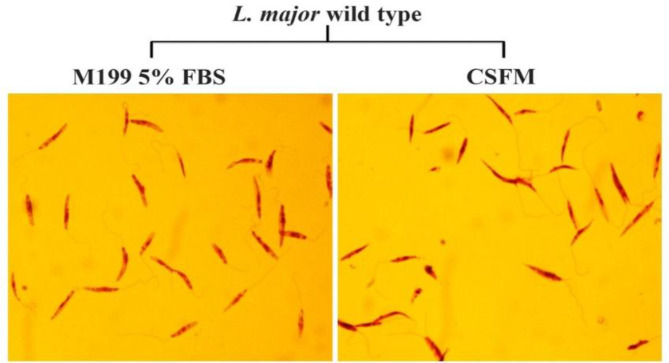
Diff-Quick staining of *L. major *parasite cultured in CSFM and M199 with 5% FBS medium. The *L. major *parasites cultured in CSFM and M199 with 5% FBS were stained at log-phase with Diff-Quick staining and observed by a light microscope at a 100× magnification

According to the results, the growth rate of *L. major *promastigotes in the CSFM medium appeared to be similar to M199 supplemented with 5% FBS. The highest cell density attained on the fourth day with the parasite number of 3.1 × 10^7^ ml^-1 ^in CSFM medium and 4.2 × 10^7^ parasites ml^-1 ^in the M199 with 5% FBS (Fig. 3A). As depicted in Fig. 3A and 3B, there were no significant differences in the level of *L. major* growth rate cultured in CSFM medium in comparison to the control group at all indicated time points (*p* > 0.05). In parallel, Diff-Quick staining displayed typical flagellated morphology of *L. major* promastigotes cultured in both media during growth analysis (Fig. 4). In the next step, the impact of CSFM medium on parasite infectivity was determined by the subcutaneous injection of *L. major* stationary phase into the left footpad of BALB/c mice, and the lesion size was measured weekly up to seven weeks along with parasite load quantification. The footpad swelling demonstrated that infection by *L. major* cultured in CSFM medium confers a considerable swelling in BALB/c mice. Approximately four weeks after infection, the swelling was observed in each group, and lesions were detected after six weeks. Based on Figure 3C, the level of swelling was similar in animals infected with *L. major *grown in both CSFM and M199 with 5% FBS, and no significant differences were detected among groups (*p* > 0.05). Also, the amount of parasite load in the lymph node of BALB/c mice infected with *L. major* promastigotes grown in CSFM and M199 with 5% FBS was assessed by real-time PCR at seven weeks post infection. Considering the absolute copy numbers of the *L. major* parasite in Figure 3D, the highest parasite propagation was detected in animals infected by parasites cultured in M199 with 5% FBS, but no significant differences were observed between these two groups (*p *> 0.05). The results obviously indicated the similar potential of *L. major *grown in CSFM medium to infect BALB/c mice, as well as parasites cultured in M199 with 5% FBS. Both parasite load and footpad swelling validated similar ability to induce infection in BALB/c mice by *L. major* parasites grown in CSFM medium and M199 with 5% FBS. 

## DISCUSSION

 The primary requirement for *Leishmania* vaccine research is a large volume production of promastigotes; therefore, liquid culture medium that facilitates this condition is an essential issue^[^^18^^]^. In this direction, there are various types of media used for *Leishmania,* which mostly require FBS as an expensive supplement and sometimes with variable qualities. Accessibility of FBS is also another issue in some parts of tropical and subtropical countries involved in leishmaniasis. There are also the risk of different contaminations in FBS, if live vaccine development is the target goal^[^^33^^]^. Several studies have been performed to replace FBS in the culture medium with other various components to reduce the aforementioned problems due to FBS^[^^17^^,^^22^^,^^34^^]^. The results of these studies demonstrated that *Leishmania* parasite is able to grow and survive in different media in the absence of FBS as good as the medium supplemented with FBS, although some of them are not suitable for therapeutic purposes and even the bulk culture of *Leishmania* promastigotes^[^^17^^,^^35^^,^^36^^]^.

 In the present study, we aimed to replace FBS with the cost-effective supplementary substances to culture and propagate the *Leishmania* parasite, especially for the cultivation of the *L. tarentolae-*PpSP15-EGFP parasite in favor of vaccine development and therapeutic purposes, which required a safe condition for production process. In this regard, to find a suitable substitution for FBS, the nutritional needs of *Leishmania* parasites were investigated. It is known that the amino acid requirements of *Leishmania *parasites are complex. Some studies have been performed in the past to determine the required amino acids for *Leishmania* promastigotes by removing each amino acid separately from the culture medium, and the results showed that the amino acids, including tryptophan, phenylalanine, arginine, leucine, lysine, valine, serine, histidine, tyrosine, and threonine, are essential for the survival and continuous cultivation of *Leishmania* promastigotes^[^^34^^,^^37^^]^. It is also known that heme is an important biomolecule acting as a critical regulatory component in oxidative metabolism, storage and transport of oxygen and signal transduction in different species of* Leishmania*, ultimately affecting the parasite growth and survival. It is already shown that *Leishmania* parasite and other protozoa, e.g. trypanosomatids, have metabolic defect and lack the required enzymes for the synthesis of this molecule. Therefore, it is necessary to obtain heme from their environment^[^^38^^,^^39^^]^. Other nutrients require for* Leishmania* parasite include vitamins such as nicotinic acid, folic acid, biotin, thiamine, riboflavin, and pantothenate^[^^40^^,^^41^^]^. 

 In the current study, a novel serum-free medium named CSFM was compared with M199 enriched with 5% FBS as commercially available and widely used liquid medium to evaluate the morphological and biological criteria of *L. tarentolae*-PpSP15-EGFP and *L. major* wild type parasites. The typical appearance of *L. tarentolae*-PpSP15-EGFP promastigotes was found to be flagellated, motile, and in normal size in CSFM medium, exactly the same as the parasites cultured in M199 with 5% FBS. Comparable results were obtained for the parasite growth rate in both CSFM and M199 with 5% FBS media. Flow cytometry analysis in this study clearly indicated that the percentage of cells expressing EGFP by *L. tarentolae*-PpSP15-EGFP parasites cultured in CSFM medium in both logarithmic and stationary growth phases were similar to the parasites grown in M199 with 5% FBS. Fluorescent microscope observations were also consistent with the flow cytometry results and indicated that the intensity of fluorescent light induced by EGFP expression by *L. tarentolae-*PpSP15-EGFP was equivalent in CSFM medium and M199 with 5% FBS medium in both logarithmic and stationary growth stages. Immunoblotting analysis displayed that the expression profile of PpSP15-EGFP protein in the *L. tarentolae*-PpSP15-EGFP promastigotes grown in CSFM medium, in terms of molecular weight and relative intensity, is similar to serum-enriched culture medium. These findings clearly demonstrated that *L. tarentolae*-PpSP15-EGFP parasites are capable of proliferating and are able to survive with the proper expression of PpSP15-EGFP in CSFM medium. We also demonstrated that the parasite grown in CSFM medium had the same ability, in comparison with M199 with 5% FBS, to infect BALB/c mice. Diff-Quick smears exhibited the normal morphological characteristics of *L. major* promastigotes grown in both media. The parasite load and foot-pad swelling measurement with a significant lesion exhibited the proper infection potency of *L. major* promastigotes in both media. Therefore, growth in this serum-free medium had no effect on typical morphological characteristics and the infectivity of the *L. major* parasite. These results clearly displayed that removal of FBS due to replacement with other nutrients in the CSFM medium had no influence on the parasite at the level of *in vivo* infectivity. 

 Altogether, our data support that the CSFM medium due to its constituents, including essential and non-essential amino acids, vitamins, high glucose, and other nutrients, is able to support the growth and infectivity of parasites for both *L. tarentolae*-PpSP15-EGFP and *L. major* wild-type parasites, respectively. The other important issue is that the CSFM has affordable ingredients with easy access and also is cost-effective. Furthermore, this culture medium has the ability to be used in the fermenter for the bulk production of *Leishmania* for vaccination and therapeutic purposes. 

## References

[1] Steverding D (2017). The history of leishmaniasis. Parasites and vectors.

[2] WHO (2020). Leishmaniasis.

[3] Kevric I, Cappel MA, Keeling JH (2015). New World and Old World Leishmania Infections. Dermatologic clinics.

[4] Ghorbani M, Farhoudi R (2018). Leishmaniasis in humans: drug or vaccine therapy?. Drug design, development and therapy.

[5] Saunders EC, Naderer T, Chambers J, Landfear SM, McConville MJ (2018). Leishmania mexicana can utilize amino acids as major carbon sources in macrophages but not in animal models. Molecular microbiology.

[6] Khademvatan S, Salmanzadeh S, Foroutan-Rad M, Bigdeli S, Hedayati-Rad F, Saki J, Heydari-Gorji E (2017). Spatial distribution and epidemiological features of cutaneous Leishmaniasis in southwest of Iran. Alexandria journal of medicine.

[7] Desjeux P (2004). Leishmaniasis: current situation and new perspectives. Comparative immunology, microbiology and infectious diseases.

[8] Kohl K, Zangger H, Rossi M, Isorce N, Lye LF, Owens KL, Beverley SM, Mayer A, Fasel N (2018). Importance of polyphosphate in the Leishmania life cycle. Microbial cell.

[9] Bates PA (2018). Revising Leishmania's life cycle. Nature microbiology.

[10] Lai JY, Klatt S, Lim TS (2019). Potential application of leishmania tarentolae as an alternative platform for antibody expression. Critical reviews in biotechnology.

[11] Doukas A, Karena E, Botou M, Papakostas K, Papadaki A, Tziouvara O, Xingi E, Frillingos S, Boleti H (2019). Heterologous expression of the mammalian sodium-nucleobase transporter rSNBT1 in Leishmania tarentolae. Biochimica et biophysica acta biomembranes.

[12] Klatt S, Simpson L, Maslov DA, Konthur Z (2019). Leishmania tarentolae: Taxonomic classification and its application as a promising biotechnological expression host. PLoS neglected tropical diseases.

[13] Abdossamadi Z, Taheri T, Seyed N, Montakhab Yeganeh H, Zahedifard F, Taslimi Y, Habibzadeh S, Gholami E, Gharibzadeh S, Rafati S (2017). Live Leishmania tarentolae secreting HNP1 as an immunotherapeutic tool against Leishmania infection in BALB/c mice. Journal of immunotherapy.

[14] Pirdel L, Farajnia S (2017). A non-pathogenic recombinant Leishmania expressing Lipophosphoglycan 3 against experimental infection with Leishmania infantum. Scandinavian journal of immunology.

[15] Katebi A, Gholami E, Taheri T, Zahedifard F, Habibzadeh S, Taslimi Y, Shokri F, Papadopoulou B, Kamhawi S, Valenzuela J, Rafati S (2015). Leishmania tarentolae secreting the sand fly salivary antigen PpSP15 confers protection against Leishmania major infection in a susceptible BALB/c mice model. Molecular immunology.

[16] Gholami E, Oliveira F, Taheri T, Seyed N, Gharibzadeh S, Gholami N, Mizbani A, Zali F, Habibzadeh S, Bakhadj DO (2019). DNA plasmid coding for Phlebotomus sergenti salivary protein PsSP9, a member of the SP15 family of proteins, protects against Leishmania tropica. PLoS neglected tropical diseases.

[17] Merlen T, Sereno D, Brajon N, Rostand F, Lemesre JL (1999). Leishmania spp: completely defined medium without serum and macromolecules (CDM/LP) for the continuous in vitro cultivation of infective promastigote forms. The American journal of tropical medicine and hygiene.

[18] de Almeida Rodrigues I, da Silva BA, dos Santos ALS, Vermelho AB, Alviano CS, Rosa MdSS (2010). A new experimental culture medium for cultivation of Leishmania amazonensis: its efficacy for the continuous in vitro growth and differentiation of infective promastigote forms. Parasitology research.

[19] Van der Valk J, Mellor D, Brands R, Fischer R, Gruber F, Gstraunthaler G, Hellebrekers L, Hyllner J, Jonker F, Prieto P, Baumans V (2004). The humane collection of fetal bovine serum and possibilities for serum-free cell and tissue culture. Toxicology in vitro.

[20] Mesalam A, Lee KL, Khan I, Chowdhury M, Zhang S, Song SH, Joo MD, Lee JH, Jin JI, Kong IK (2019). A combination of bovine serum albumin with insulin–transferrin–sodium selenite and/or epidermal growth factor as alternatives to fetal bovine serum in culture medium improves bovine embryo quality and trophoblast invasion by induction of matrix metalloproteinases. Reproduction, fertility and development.

[21] Barnes D, Sato G (1980). Methods for growth of cultured cells in serum-free medium. Analitical biochemistry metods in the biological sciences.

[22] Rauch C, Feifel E, Amann E-M, Spötl HP, Schennach H, Pfaller W, Gstraunthaler G (2011). Alternatives to the use of fetal bovine serum: human platelet lysates as a serum substitute in cell culture media. Alternatives to animal experimentation.

[23] Brunner D, Frank J, Appl H, Schöffl H, Pfaller W, Gstraunthaler G (2010). The serum-free media interactive online database. Alternatives to animal experimentation.

[24] Gstraunthaler G (2003). Alternatives to the use of fetal bovine serum: serum-free cell culture. Alternatives to animal experimentation.

[25] Barnes D, Sato G (1980). Serum-free cell culture: a unifying approach. Cell.

[26] Muniaraj M, Lal C, Kumar S, Sinha P, Das P (2007). Milk of cow (Bos taurus), buffalo (Bubalus bubalis), and goat (Capra hircus): a better alternative than fetal bovine serum in media for primary isolation, in vitro cultivation, and maintenance of Leishmania donovani promastigotes. Journal of clinical microbiology.

[27] Belford DA, Rogers ML, Regester GO, Francis GL, Smithers GW, Liepe IJ, Priebe IK, Ballard FJ (1995). Milk-derived growth factors as serum supplements for the growth of fibroblast and epithelial cells. In vitro cellular and developmental biology-animal.

[28] Sidana A, Alam A, Farooq U (2018). Soy protein isolate: A substitute of fetal bovine serum for the in vitro cultivation of Leishmania donovani. Legume research-an international journal.

[29] Fritsche C, Sitz M, Weiland N, Breitling R, Pohl HD (2007). Characterization of the growth behavior of Leishmania tarentolae–a new expression system for recombinant proteins. Journal of basic microbiology.

[30] Sharief AH, Khalil EAG, Omer SA, Abdalla HS (2008). Innovative serum-free medium for in vitro cultivation of promastigote forms of Leishmania species. Parasitology international.

[31] Ali SA, Iqbal J, Ahmad B, Masoom M (1998). A semisynthetic fetal calf serum-free liquid medium for in vitro cultivation of Leishmania promastigotes. The American journal of tropical medicine and hygiene.

[32] Bolhassani A, Taheri T, Taslimi Y, Zamanilui S, Zahedifard F, Seyed N, Torkashvand F, Vaziri B, Rafati S (2011). Fluorescent Leishmania species: development of stable GFP expression and its application for in vitro and in vivo studies. Experimental parasitology.

[33] Nasiri V (2017). An overview of the recent findings in the cultivation of Leishmania. Reviews in medical microbiology.

[34] Trager W (1957). Nutrition of a hemoflagellate (Leishmania tarentolae) having an interchangeable requirement for choline or pyridoxal. The journal of protozoology.

[35] Kar K, Mukerji K, Naskar K, Bhattacharya A, Ghosh DK (1990). Leishmania donovani: a chemically defined medium suitable for cultivation and cloning of promastigotes and transformation of amastigotes to promastigotes. The journal of protozoology.

[36] Van der Valk J, Brunner D, De Smet K, Svenningsen AF, Honegger P, Knudsen LE, Lindl T, Noraberg J, Price A, Scarino M (2010). Optimization of chemically defined cell culture media–replacing fetal bovine serum in mammalian in vitro methods. Toxicology in vitro.

[37] Nayak A, Akpunarlieva S, Barrett M, Burchmore R (2018). A defined medium for Leishmania culture allows definition of essential amino acids. Experimental parasitology.

[38] Laranjeira-Silva MF, Hamza I, Pérez-Victoria JM (2020). Iron and Heme Metabolism at the Leishmania–Host Interface. Trends in Parasitology.

[39] Toh SQ, Glanfield A, Gobert GN, Jones MK (2010). Heme and blood-feeding parasites: friends or foes?. Parasites and vectors.

[40] Schuster FL, Sullivan JJ (2002). Cultivation of clinically significant hemoflagellates. Clinical microbiology reviews.

[41] Santarem N, Cunha J, Silvestre R, Silva C, Moreira D, Ouellette M, Cordeiro-Da-Silva A (2014). The impact of distinct culture media in Leishmania infantum biology and infectivity. Parasitology.

